# Motif-Independent Prediction of a Secondary Metabolism Gene Cluster Using Comparative Genomics: Application to Sequenced Genomes of *Aspergillus* and Ten Other Filamentous Fungal Species

**DOI:** 10.1093/dnares/dsu010

**Published:** 2014-04-11

**Authors:** Itaru Takeda, Myco Umemura, Hideaki Koike, Kiyoshi Asai, Masayuki Machida

**Affiliations:** 1 Department of Biotechnology and Life Science, Tokyo University of Agriculture and Technology, 2-24-16 Naka-cho, Koganei, Tokyo 184-8588, Japan; 2 Bioproduction Research Institute, National Institute of Advanced Industrial Science and Technology (AIST), Higashi 1-1-1, Tsukuba, Ibaraki 305-8566, Japan; 3 Bioproduction Research Institute, National Institute of Advanced Industrial Science and Technology (AIST), Higashi-Nijo 17-2-1, Tsukisamu, Sapporo, Hokkaido 062-8517, Japan; 4 Department of Computational Biology, Graduate School of Frontier Sciences, The University of Tokyo, 5-1-5 Kashiwanoha, Kashiwa-shi Chiba 277-8561, Japan; 5 Computational Biology Research Center (CBRC), National Institute of Advanced Industrial Science and Technology (AIST), Tokyo Waterfront Bio-IT Research Building, 2-4-7 Aomi, Koto-ku, Tokyo 135-0064, Japan

**Keywords:** secondary metabolism, bioinformatics, filamentous fungi

## Abstract

Despite their biological importance, a significant number of genes for secondary metabolite biosynthesis (SMB) remain undetected due largely to the fact that they are highly diverse and are not expressed under a variety of cultivation conditions. Several software tools including SMURF and antiSMASH have been developed to predict fungal SMB gene clusters by finding core genes encoding polyketide synthase, nonribosomal peptide synthetase and dimethylallyltryptophan synthase as well as several others typically present in the cluster. In this work, we have devised a novel comparative genomics method to identify SMB gene clusters that is independent of motif information of the known SMB genes. The method detects SMB gene clusters by searching for a similar order of genes and their presence in nonsyntenic blocks. With this method, we were able to identify many known SMB gene clusters with the core genes in the genomic sequences of 10 filamentous fungi. Furthermore, we have also detected SMB gene clusters without core genes, including the kojic acid biosynthesis gene cluster of *Aspergillus oryzae*. By varying the detection parameters of the method, a significant difference in the sequence characteristics was detected between the genes residing inside the clusters and those outside the clusters.

## 1. Introduction

Secondary metabolites are an important resource for bioactive compounds, including lead compounds for new drugs, effective components of functional foods and chemical raw materials. Although a variety of secondary metabolites have been discovered primarily from actinomycetes, fungi and plants, a significantly larger number of secondary metabolites are thought to remain undetected due to the silencing of corresponding biosynthesis genes under the conditions used for screening.^1–3^

The genes responsible for the biosynthesis of each secondary metabolite are often clustered in the genome.^4^ Furthermore, the basic structures of the known secondary metabolites are often synthesized by the so-called core genes, polyketide synthase (PKS), nonribosomal peptide synthetase (NRPS) and dimethylallyltryptophan synthase (DMAT). Thus, BLAST and Pfam searches for domains in polypeptides encoded by these genes have served as powerful means in identifying essential genes in secondary metabolism biosynthesis (SMB) gene clusters. Clust Scan and CLUSEAN identify core genes for SMB by searching the functional domains and motifs of PKS and NRPS.^4,5^ Other software tools, such as SMURF and antiSMASH, first identify the core genes using their motifs and then extend the flanking genes with homology to genes frequently found in the known SMB gene clusters, including hydroxylases, oxidases, methylases, transcription factors (typically Zn(II)Cys6 binuclear cluster types) and transporter genes.^6,7^ However, some SMB gene clusters, such as the oxylipin^8^ and kojic acid^9^ biosynthesis gene clusters, lack core genes in their clusters. These examples indicate the importance of devising a method for the prediction of SMB gene clusters without using the known motifs of the core genes.

Recently, the development of next-generation sequencing technology has dramatically accelerated the sequencing of the genomes of diverse organisms. Even the genomes of filamentous fungi, which have relatively large genome sizes among microbes, can be accurately sequenced without reference genomes.^10^ The extremely high throughput and low cost of sequencing have increased the motivation to sequence the genomes of closely related species and even strains of the same species^11^ for detailed and comprehensive genome comparisons.

In this study, we developed a novel method that applies a comparative genomics approach to predict SMB gene clusters, including those without core genes. This method depends on the characteristics of secondary metabolism genes, namely that they are highly enriched in non-syntenic blocks^12^ and are rarely orthologous even between clusters producing similar compounds due to generally high sequence diversity.^13^ Our method successfully predicted SMB gene clusters without using motif information from known genes in the SMB gene clusters. Through the optimization of the prediction parameters, we have also depicted the structural characteristics of the SMB gene clusters.

## 2. Materials and methods

### 2.1. Genome data

The nucleotide and amino acid sequences of the genomes and deduced coding sequences, respectively, were retrieved from the following databases: *Aspergillus flavus* (accession no. EQ963472∼EQ963493) and *A. oryzae* (accession no. AP007150∼AP007177) from DDBJ/EMBL/GenBank DNA database; *A. fumigatus*, *A. nidulans* and *A. terreus* from the *Aspergillus* comparative database (http://www.broadinstitute.org/annotation/genome/aspergillus_group/MultiHome.html); *Magnaporthe grisea* from the *Magnaporthe* comparative database (http://www.broadinstitute.org/annotation/genome/magnaporthe_comparative/MultiHome.html); *Chaetomium globosum* from the *Chaetomium globosum* database (http://www.broadinstitute.org/annotation/genome/chaetomium_globosum); and *Fusarium graminearum*, *F. oxysporum* and *F. verticillioides* from the *Fusarium* comparative database (http://www.broadinstitute.org/annotation/genome/fusarium_group/MultiHome.html) at The Broad Institute**.** The gene IDs of GenBank was assigned to the genes annotated by Broad Institute by using BLASTP search.

### 2.2. Algorithm overview

**Figure 1.**
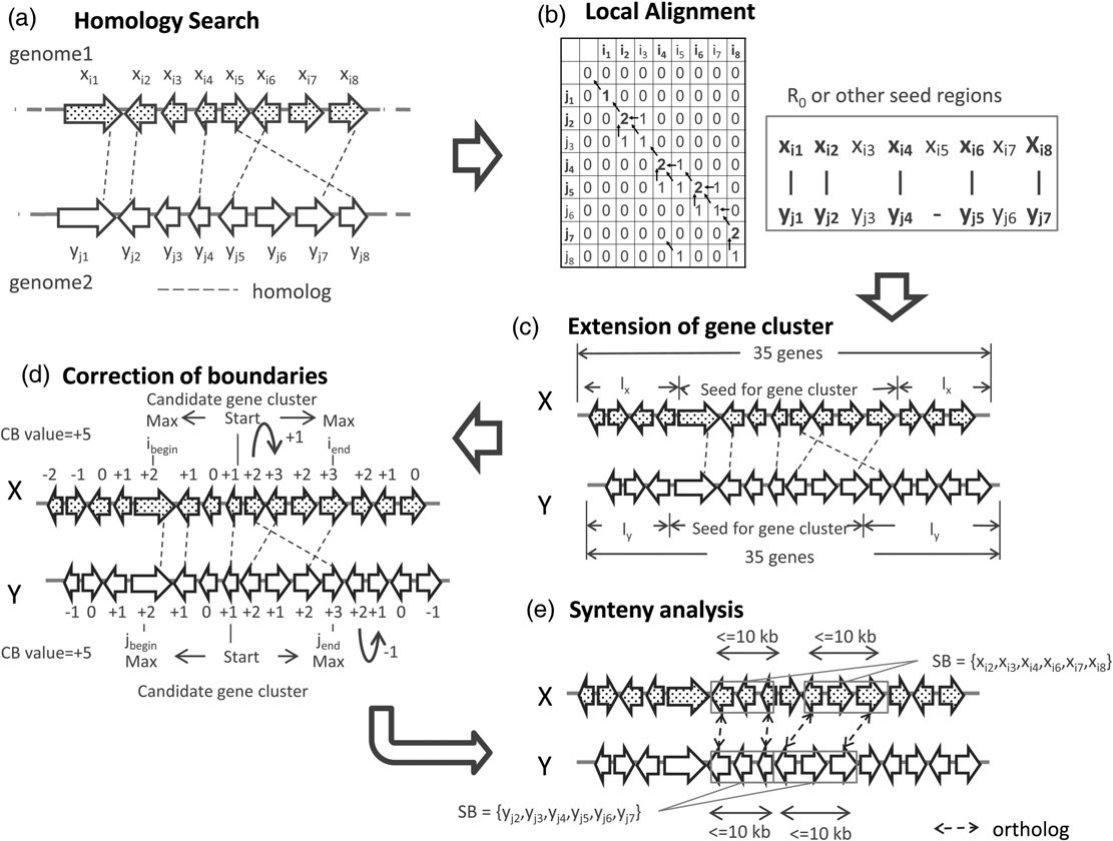
Overview of the prediction method for SMB gene clusters. (a) Broken lines represent homologous gene pairs between two genomes. Each pair of ‘*x_i_*_1_’–‘*y_j_*_1_’, ‘*x_i_*_2_’–‘y_j2_’, ‘*x_i_*_4_’–‘*y_j_*_4_’, ‘*x_i_*_5_’–‘*y_j_*_8_’, ‘*x_i_*_6_’–‘*y_j_*_5_’ and ‘*x_i_*_8_’–‘*x_j_*_7_’ represents a homolog. The *x_i_* and *y_j_* represent genes in the first and the second genomes, respectively. (b) The genes were aligned in the genome using the Smith–Waterman algorithm (Param2 = −1). Pairs of contiguous genes from ‘*x_i_*_1_’ to ‘*x_i_*_8_’ in genome 1 and from ‘*y_j_*_1_’ to ‘*y_j_*_7_’ represent an example identified as a seed for predicting a gene cluster (*R*_0_ or other seed regions). (c) The seed was extended until the prescribed length (Param3 = 35). The symbols *l_x_* and *l_y_* represent the numbers of genes added to the seed region of the first and the second genomes, respectively. *X* and *Y* represent extended clusters in the first and the second genomes, respectively. (d) The boundaries were corrected (Param4 = −1), and a pair of candidate gene clusters, ‘*x_i_*_1_’ through ‘*x_i_*_8_’ and ‘*y_j_*_1_’ through ‘*y_j_*_8_’, was identified. The symbols *i*_begin_ and *i*_end_ represent the locations of the genes at the beginning and end, respectively, of the cluster in the first genome. The symbols *j*_begin_ and *j*_end_ represent the corresponding gene locations in the second genome. The CB value is the sum of the maximum scores for the upstream and the downstream boundaries of a predicted cluster. The integers are indicated as an example for the particular alignment of clusters represented in this figure. (e) Synteny analysis was performed to distinguish the SMB gene cluster from the syntenic block (SB). The SB, a subset of *X* and *Y*, represents a set of genes aligned to create a contiguous block of orthologous gene pairs located within the defined distance between neighboring genes (Param5 = 10 kb). The above parameters are examples and not necessarily those used for the actual analyses.

The method for prediction of gene clusters devised in this study consists of three steps. The first step is to search pairwise similarity between the genes in the two genomes and to perform successive alignment detections of homologous genes (Fig. 1a and b). This step is based on the assumption that SMB gene clusters that produce compounds that are not identical but that have common basic structures have similar member genes. Low gap and mismatch penalties allow the detection of a gene cluster pair containing inversions and/or deletions in their gene members. The second step is to correct the boundary of the predicted gene cluster. This step is achieved by scoring homologous genes, considering genes outside but proximal to the predicted gene cluster (Fig. 1c and d). The third step is to enrich gene clusters with higher probability to function as SMB gene clusters via synteny analysis (Fig. 1e). Secondary metabolism genes are highly enriched on nonsyntenic blocks when the *A. oryzae* genome is compared with the genome of *A. fumigatus* or *A. nidulans*.^12^ Thus, of the gene clusters predicted in the prior step, those forming syntenic blocks can be eliminated (Fig. 1e).

### 2.3. Identification of homologous and orthologous gene pairs

Prior to comparing the order of genes between a pair of genomes, homologous gene pairs were identified in an amino acid homology search (Fig. 1a) using BLASTP^14,15^ with e-values (Param1) of 1.0e−5, 1.0e−10, 1.0e−15, 1.0e−30 or 1.0e−50 as thresholds. Orthologs were determined using the bidirectional best BLASTP hit method.

### 2.4. Identification of the seed region pair for a gene cluster

The regions for which the order of genes was conserved between the genome pair were searched by local alignment of the genes with the Smith–Waterman algorithm^16,17^ (Fig. 1b). The genes in the first and second genomes were defined as *x_i_* (*i* = 1, 2, …, *I*) and *y_j_* (*j* = 1, 2, … , *J*), respectively. A matrix (SW) of (*J* + 1) × (*I* + 1) was prepared by calculating each cell score according to the following formulas:

$$\text{SW}(\;j,i)=max\left\{\begin{array}{c} \text{SW}(\;j-1,i-1)+1\\ \text{SW}(\;j,i-1)+P_{\mathrm{gap}}\\ \text{SW}(\;j-1,i)+P_{\mathrm{gap}}\\ 0 \end{array}\right.$$

when a pair of genes, *x_i_* and *y_j_*, are homologous.

$$\text{SW}(\;j,i)=max\left\{\begin{array}{c} \text{SW}(\;j-1,i-1)+P_{\mathrm{mismatch}}\\ \text{SW}(\;j,i-1)+P_{\mathrm{gap}}\\ \text{SW}(\;j-1,i)+P_{\mathrm{gap}}\\ 0 \end{array}\right.$$

when *x_i_* and *y_j_* are not homologous.

Values of −0.1, −0.2, −0.4, −0.5 or −1 were used for *P*_gap_ and *P*_mismatch_, a gap and a mismatch penalty, respectively (Param2). After the scores were calculated for all of the cells in the matrix based on the similarity between any gene pair, the gene cluster coordinates were obtained by tracing the cells from the pair with the maximum score to that with a score of 0 (Fig. 1b). The pair of gene cluster coordinates was defined as *R*_0_, which was used as one of the seeds for the predicted gene clusters.

$$R_{0}=\{(\;j_{1},i_{1}),(\;j_{2},i_{2}),\ldots (\;j_{m},i_{n})\},\;where\;\;j_{1}\leq \;j_{2}\leq \cdots \leq \;j_{m},i_{1}\leq i_{2}\leq \cdots \leq i_{n}$$

Other seeds were detected by a traceback of the same score matrix (see Supplementary Fig. S1). These seeds were subjected to the correction of boundaries in the next step. Gene cluster coordinates were also searched using the reverse orientation for one of the two genomes.

### 2.5. Correcting gene cluster boundaries

*R*_0_ and other seed regions, consisting of the genes *x_i_* (*i*_1_ ≤ *i* ≤ *i_n_*) and *y_j_* (*j*_1_ ≤ *j* ≤ *j_m_*), may not have the correct boundaries as a gene cluster for various reasons, particularly when part of the cluster has a reversed orientation in the gene order. This reversal could be caused by a small inversion.

In this study, by taking the actual, experimentally confirmed sizes of the clusters into consideration, the minimum number of homologous gene pairs was set to 3, and the values 15, 25, 35, 45, 55 and 65 were used for the maximum number of genes contained in a gene cluster (Param3).

After the detection of seeds with cluster sizes under the threshold, the same number of genes located in the vicinity of the seeds was added to both ends of the seeds to extend the cluster size to a predefined number of genes for successive boundary corrections (Fig. 1c). If the number of genes to be added was odd, an additional gene was added to either of the two ends of the cluster. In this study, the same values derived with Param3 were used as the cluster sizes after the addition. When 35 genes were applied to Param3, each set of genes that extended the seed in the first and the second genomes was defined as *X* and *Y*, respectively, and each number of genes added to the seed was defined as *l_x_* and *l_y_*, respectively:

$$X=\{x_{i}|i:integer\;and\;satisfying\;i_{1}-l_{x}\leq i\leq i_{n}+l_{x},\;where\;i_{n}-i_{1}+2l_{x}+1=35\}$$

$$Y=\{y_{j}|j:integer\;and\;satisfying\;\;j_{1}-l_{y}\leq j\leq \;j_{m}+l_{y},\;where\;\;j_{m}-\;j_{1}+2l_{y}+1=35\}$$

To correct the boundaries of a seed of clustered genes, homologous genes were scored from the gene located at the center of the cluster to both ends of the cluster. A score, SC, was calculated for each gene member in *X* according to the following formulas (Fig. 1d):

(1) $$\begin{array}{r} \text{SC}(i)=\left\{\begin{array}{cc} 1, & i=(i_{1}+i_{n})\text{/}2\\ \text{SC}(i+1)+1, & \;\;i<(i_{1}+i_{n})\text{/}2\;\\ \text{SC}(i-1)+1, & i>(i_{1}+i_{n})\text{/}2 \end{array}\right. \end{array}$$

when *x_i_* has at least one homolog among the members of *Y* and

(2) $$\text{SC}(i)=\left\{\begin{array}{cc} P_{\mathrm{negative}}, & i=(i_{1}+i_{n})/2\\ \text{SC}(i+1)+P_{\mathrm{negative}}, & i<(i_{1}+i_{n})/2\\ \text{SC}(i-1)+P_{\mathrm{negative}}, & i>(i_{1}+i_{n})/2 \end{array}\right.$$

when *x_i_* has no homologs among the members of *Y*.

*P*_negative_ represents a penalty score for the gene that has no homologs in the paired extended seed. Based on the scores for all of the member genes, a gene cluster candidate was defined between the genes (*i*_begin_ and *i*_end_) with the maximum scores in the regions indicated by (1–4), respectively. To evaluate similarity between a pair of detected clusters, a CB value was defined as the sum of the maximum scores at both ends. The boundary correction *Y* was determined in the same manner. Consequently, a pair of gene cluster candidates, *x_i_* and *y_j_*, was defined as follows:

$$x_{i}(i_{\mathrm{begin}}\leq i\leq i_{\mathrm{end}}),\;y_{j}\;(\;j_{\mathrm{begin}}\leq j\leq \;j_{\mathrm{end}})$$

In this study, the values −0.1, −0.2, −0.3, −0.4, −0.5 and −1 were used as the negative penalty (Param4).

### 2.6. Synteny analysis

Secondary metabolism genes are highly enriched in nonsyntenic blocks.^12^ Secondary metabolism genes, which have high sequence diversity in general,^13^ are rarely orthologous in the comparison of genomes between two species. In contrast, syntenic blocks, in which genes existing across species commonly accumulate, have a high proportion of orthologs. Thus, candidate gene clusters that have a high probability of secondary metabolism biosynthesis genes can be selected by referring to their localization in nonsyntenic blocks (Fig. 1e).

Orthologous gene pairs between *X* and *Y* were aligned to create contiguous blocks until no more orthologs were identified within the threshold range of the intergenic distances in both genomes (Fig. 1e). Contiguous blocks composed of at least two orthologs were defined as syntenic blocks (SBs). Non-orthologous genes inserted between orthologs were allowed within the threshold of an intergenic distance of 5, 10, 20, 30, 40 or 50 kb (Param5).

SBs were subsets of extended seeds, *X* and *Y*. If the percentage of the member genes in the subset segment for the number of genes in the entire extended cluster was less than the threshold, the corresponding candidate gene cluster was selected as a predicted secondary metabolism gene cluster. In this study, 10, 15, 20, 25, 30 and 35% were used as the thresholds for the SB percentage (Param6). Multiple predicted clusters overlapping each other were merged into a single cluster similarly to methods used in other SMB gene cluster prediction software.^6,18^

## 3. Results and discussion

### 3.1. Effect of each parameter on the prediction

**Figure 2.**
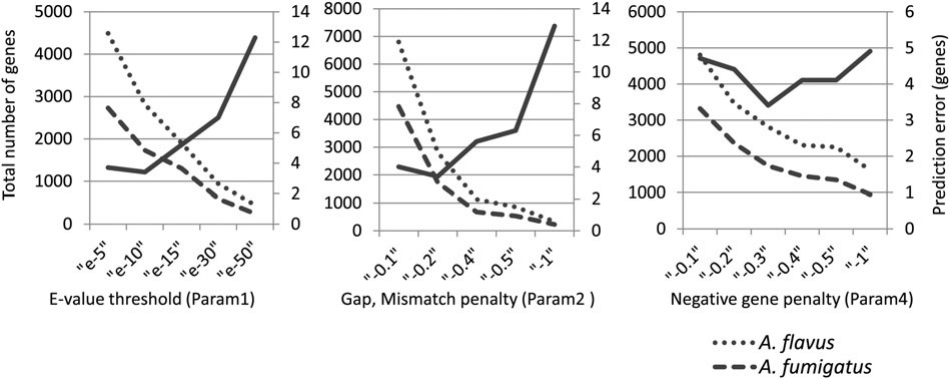
Analysis of the average prediction errors. Averages of the minimum error for predicting the known gene clusters (solid line) of *A. flavus* and *A. fumigatus* are shown together with the total numbers of genes in the predicted gene clusters of *A. flavus* (dotted line) and *A. fumigatus* (broken line). The default values, except for the parameter indicated in each panel, were the same as those used in Fig. 3.

To detect SMB gene cluster candidates, the genome sequences of 10 species of filamentous fungi, including *A. oryzae* (see Materials and methods), were subjected to a comprehensive pairwise comparison, with the exception of between identical genomes. We first detected known SMB gene clusters from the genomes of *A. flavus* and *A. fumigatus* to optimize the parameters of our method. The clusters that the method predicted for the biosynthesis of aflatoxin and gliotoxin from *A. flavus* and for the biosynthesis of ergot, epipolythiodioxopiperazine-type toxin (ETP), fumitremorgin, gliotoxin, melanin, Pes1, pseurotin and siderophore from *A. fumigatus*, are listed in Table 1 and were subjected to the analysis of differences in boundary positions when compared with those from the experimentally confirmed clusters. Absolute values of the differences in gene numbers at the upstream and the downstream boundaries were summed to generate a value defined as the prediction error. The minimum error was obtained from all of the clusters predicted for each gene cluster, and the average of the minimum errors for the 10 gene clusters from *A. flavus* and *A. fumigatus* described above was then calculated at each value for the parameters. As shown in Fig. 2, a combination including Param1 = e−10, Param2 = −0.2 and Param4 = −0.3 gave the smallest errors for the prediction of the cluster boundaries. Param3 (extension length), Param5 (intergenic distance) and Param6 (permissible ratio of syntenic blocks) had little influence on the prediction of gene clusters within the range used in this study. Consequently, Param3 = 35 genes, Param5 = 10 kb and Param6 = 25% were used.

**Table 1.** Known gene clusters predicted using this method

Predicted gene cluster		Product	Verified cluster size (genes)	Species	vs. Species (top hit)^a^	Number of hits^b^	Min difference^c^		Min error^d^	Error^d^ at the max CB valve	Max error^d^
Begin	End						Up	Down			
AFLA_139060	AFLA_139460	Aflatoxin	29	*A. flavus*	*M. grisea*	9	9	2	11	16	25
AFLA_064360	AFLA_064590	Gliotoxin	33	*A. flavus*	*A. fumigatus*	8	−3	−6	9	9	25
AO090113000131	AO090113000147	Kojic acid	3	*A. oryzae*	*A. flavus*	1	4	9	13	13	13
ANID_01036	ANID_01029	Asperfuranone	8	*A. nidulans*	*A. terreus*	8	0	0	0	0	2
–	–	Asperthecin	3	*A. nidulans*	–	0	–	–	–	–	–
ANID_02625	ANID_02624	Penicillin	6	*A. nidulans*	*A. terreus*	1	0	−3	3	3	3
ANID_07805	ANID_07825	Sterigmatocystin	25	*A. nidulans*	*A. terreus*	6	−1	0	1	1	34
ANID_08517	ANID_08524	Terrequinone	7	*A. nidulans*	*F. graminearum*	4	−4	5	9	14	15
Afu2g17960	Afu2g18040	Ergot	11	*A. fumigatus*	*A. terreus*	1	0	−2	2	2	2
Afu3g12890	Afu3g12960	ETP^e^	8	*A. fumigatus*	*A. nidulans*	4	0	0	0	0	4
Afu8g00170	Afu8g00260	Fumitremorgin	10	*A. fumigatus*	*A. oryzae*	6	0	0	0	1	5
Afu6g09610	Afu6g09740	Gliotoxin	12	*A. fumigatus*	*F. oxysporum*	8	2	0	2	4	12
Afu2g17490	Afu2g17610	Melanin	8	*A. fumigatus*	*F. graminearum*	4	4	1	5	5	11
–	–	Pes1	2	*A. fumigatus*	–	0	–	–	–	–	–
Afu8g00450	Afu8g00580	Pseurotin	6	*A. fumigatus*	*F. verticillioides*	6	8	0	8	19	19
Afu3g03350	Afu3g03480	Siderophore	13	*A. fumigatus*	*F. graminearum*	9	0	1	1	2	13
ATEG_09957	ATEG_09977	Lovastatin	17	*A. terreus*	*A. oryzae*	9	1	3	4	8	9
FGSG_02322	FGSG_02330	Aurofusarin	11	*F. graminearum*	*A. terreus*	7	−2	0	2	5	7
FGSG_02392	FGSG_02400	Zearalenone	5	*F. graminearum*	*A. terreus*	2	3	−3	6	7	7
FVEG_03384	FVEG_03379	Bikaverin	6	*F. verticillioides*	*C. globosum*	3	0	0	0	5	9
FVEG_00329	FVEG_00316	Fumonisin	16	*F. verticillioides*	*A. fumigatus*	4	0	−2	2	2	9
–	–	Fusaric acid	5	*F. verticillioides*	–	0	–	–	–	–	–
FVEG_11079	FVEG_11086	Fusarin	9	*F. verticillioides*	*M. grisea*	9	−1	0	1	3	4
FVEG_03698	FVEG_03695	Perithecium pigment	6	*F. verticillioides*	*A. flavus*	4	−2	0	2	2	3

^a^Species used for comparison when the gene cluster was detected with minimum error.

^b^Number of predicted clusters in a comprehensive pairwise comparison of the 10 genomes.

^c^Difference in the numbers of genes upstream and downstream of the predicted gene cluster compared with the experimentally characterized cluster. The minus and plus quantities indicate under- and over-predictions, respectively.

^d^Error is defined as the sum of absolute values of the differences in gene number at both ends of a predicted gene cluster.

^e^Epipolythiodioxopiperazine-type toxin.

**Figure 3.**
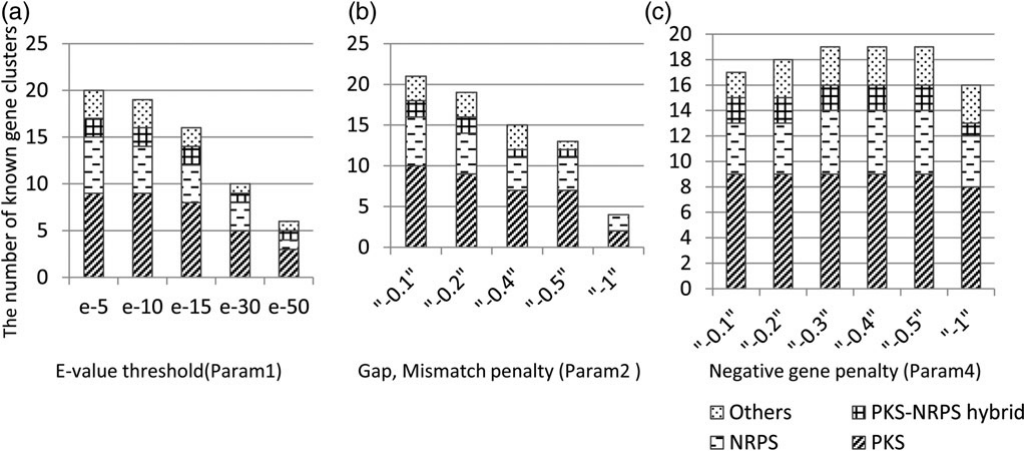
Prediction of known gene clusters. The numbers of known gene clusters that were predicted within the minimum error of 10 genes were analyzed by varying three parameters, Param1, Param2 and Param4, one by one in a, b and c, respectively. The tentative default values, except for the parameter indicated in each panel, were Param1 = e−10, Param2 = −0.2, Param3 = 35 genes, Param4 = −0.3, Param5 = 10 kb and Param6 = 25%.

**Figure 4.**
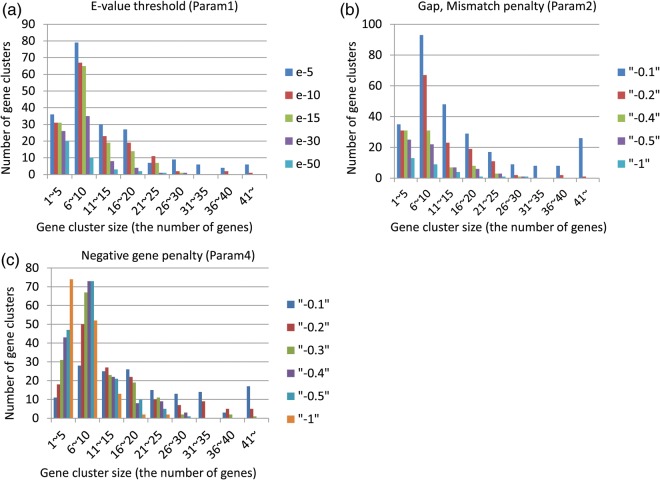
Prediction of *Aspergillus fumigatus* gene clusters. The number of predicted gene clusters (*Y* axis) in the indicated size range (*X* axis) was analyzed by varying Param1, Param2 and Param4 using the results of *A. fumigatus* as an example. The default values, except for the parameter indicated in each panel, were the same as those used in Fig. 3.

To evaluate the performance of our method, we detected known SMB gene clusters using the genomes of 10 filamentous fungal species. Of the 24 gene clusters that have been identified to date, together with their corresponding products (Supplementary Table S1), 21 gene clusters were successfully detected with the optimized parameters described above (Table 1). The minimum and the maximum errors among all of the predicted gene clusters and the error for the cluster with the maximum CB value are also indicated. Figure 3 shows the effects of Param1, Param2 and Param4 on the number of known SMB gene clusters that were detected within the minimum error of 10 genes. The number of clusters increased by decreasing the stringency of Param1 and Param2 simply because of the increased sensitivity for seed detection. A similar increase in the detected clusters was observed in the detection performed with the *A. fumigatus* genome regardless of whether the clusters were previously known (Fig. 4a and b). In contrast, decreasing the stringency of Param4 resulted in a decrease in the number of detected clusters (Fig. 3c). This result was also observed for the detection of clusters with fewer member genes, i.e. <10 (Fig. 4c). Some gene clusters located within a short distance of each other in the genome were predicted as a merged single cluster of genes when a low stringency for Param4 was given. Accordingly, this low stringency led to an increase in the number of clusters with large cluster sizes (Fig. 4c).

Although Param2 and Param4 are penalties for the alignment of homologous genes, the former takes the order of the genes into consideration, but the latter does not. A decrease in the number of predicted clusters for a stringent Param2 and little change on the number by Param4, as shown above, indicated that a pair of gene clusters has similar gene contents in terms of sequence similarity, although the order of the genes might be partially rearranged, such as by inversion.

### 3.2. Detailed analysis of successful and failed predictions of known gene clusters

Of the 21 known SMB gene clusters predicted using our method (Table 1), some of the clusters were predicted by comparing two genomes belonging to different genera. For example, the gene clusters for the biosynthesis of aflatoxin in *A. flavus* and fumonisin in *F. verticillioides* were predicted by comparison with the *M. grisea* and *A. fumigatus* genomes, respectively. The SMB gene clusters appear to be composed of genes with common sequence characteristics, even between genomes from different species with phylogenetically extensive distances.

Despite the high probability of detecting the known SMB gene clusters described above, the detection of clusters for Pes1, fusaric acid and asperthecin failed. The Pes1 and asperthecin biosynthesis gene clusters consisted of only two and three genes, respectively, and had little or no chance of having conserved homologous pairs longer than three genes in the same order in the genome. The fusaric acid biosynthesis gene cluster, which contains a total of five genes, included three genes that had unique sequences. Given the abovementioned reasons, ∼12.5% of the known SMB gene clusters are thought to remain unpredicted. The kojic acid biosynthesis gene cluster, which consisted of three genes with only weak similarity to the genes sequenced to date, was successfully detected, although its cluster size was overestimated. It is thought that the existence of genes adjacent to a cluster with a high similarity to genes of a distantly related gene cluster led to the successful detection of this short gene cluster (Table 1).

Considering the accuracy of detecting the known SMB gene clusters described above, the predicted unknown gene clusters without the core genes are highly likely to also be involved in SMB (see Supplementary Tables S2–S5, complete lists of predicted clusters from *A. nidulans*, *A. fumigatus, A. flavus* and *A. oryzae*). To further evaluate the probability of a relationship with SMB, the content of Q (secondary metabolism) genes in the euKaryotic Orthologous Groups (KOG) functional category was analyzed. The ratios of the Q genes in the predicted clusters and the remainder of the genes on nonsyntenic blocks from the *A. fumigatus* genome were 119/1,038 and 100/2,297, respectively. The successive statistical analysis of this result indicated enrichment for Q genes in the predicted clusters with a *P*-value of 10^−13^, which strongly suggested that the predicted unknown gene clusters were related to SMB regardless of the existence of core genes in the cluster.

**Figure 5.**
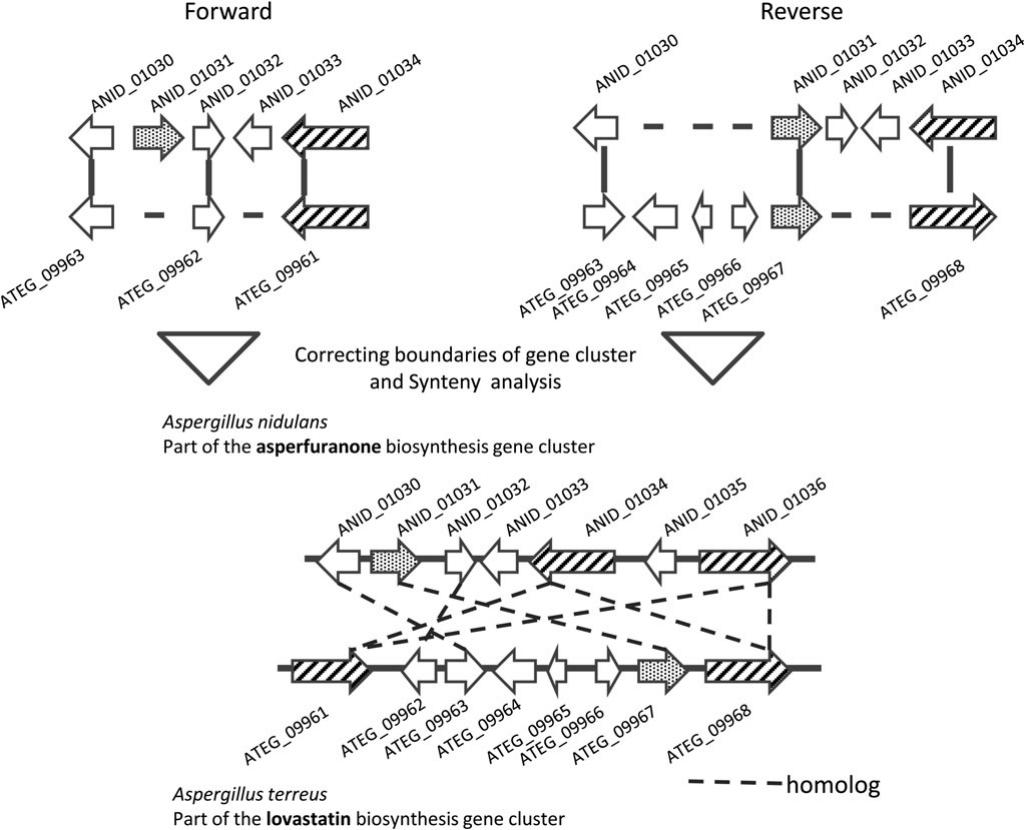
Schematic drawing of an example of a predicted known SMB gene cluster. The top figures represent seeds used in the detection of a pair of SMB gene clusters for *A. nidulans* asperfuranone and *A. terreus* lovastatin. The left and the right panels show the alignments in the forward and reverse directions, respectively. The bottom figure shows all of the homologous gene pairs included between the two clusters. No orthologs were identified in this pair of gene clusters.

Interestingly, some known gene clusters were detected by comparison with the gene cluster that appeared to have little relationship except for the core structure of the products, such as polyketide, a nonribosomal peptide. For example, the *A. nidulans* asperfuranone biosynthesis and *A. terreus* lovastatin biosynthesis gene clusters (Fig. 5, Table 2) consisted of genes annotated as PKS, oxidoreductase and a transporter (Table 2). These gene clusters were aligned in the forward and reverse directions to create a seed (Fig. 5).

**Table 2.** Examples of member genes in a predicted gene cluster

GID	Protein	Predicted function	GID	Protein^a^	Predicted function	E-value^b^
ANID_01030	406 aa	Zinc-binding oxidoreductase	ATEG_09963	364 aa	hypothetical protein similar to enoyl reductase	2.00E−18
ANID_01031	564 aa	MFS transporter	ATEG_09967	543 aa	hypothetical protein similar to efflux pump	7.00E−97
ANID_01032	298 aa	Conserved hypothetical protein	ATEG_09962	257 aa	hypothetical protein similar to oxidoreductase	7.00E−12
ANID_01034	2723 aa	Polyketide synthase	ATEG_09961	3005 aa	hypothetical protein similar to polyketide synthase	6.00E−57
ANID_01034	2723 aa	Polyketide synthase	ATEG_09968	2453 aa	hypothetical protein similar to polyketide synthase	5.00E−34
ANID_01036	2528 aa	Polyketide synthase	ATEG_09968	2543 aa	hypothetical protein similar to polyketide synthase	0.00E+00
ANID_01036	2528 aa	Polyketide synthase	ATEG_09961	3005 aa	hypothetical protein similar to polyketide synthase	0.00E+00

^a^Length of the polypeptide in amino acids.

^b^E-value of the similarity between the proteins of the detected gene clusters.

### 3.3. Properties of secondary metabolism genes

We devised a comparative genomics method for predicting SMB gene clusters by effectively utilizing the rapidly growing accumulation of genome sequences. In this study, we have successfully identified the known SMB gene clusters with a high probability (21 out of 24 clusters). The results indicate overall similarities in the amino acid sequences and/or the order of member genes between the pairs of gene clusters, including those involved in the biosynthesis of *A. nidulans* asperfuranone and *A. terreus* lovastatin, *A. fumigatus* fumitremorgin and *F. verticillioides* fumonisin and *A. fumigatus* melanin and *F. graminearum* aurofusarin.

Secondary metabolism genes are highly enriched in the nonsyntenic blocks in a comparison of the genomes of three *Aspergillus* species.^12^ We have applied this observation to our method and have successfully identified various known SMB gene clusters from the genomes of the 10 fungal species, including those outside the genus *Aspergillus*. This observation indicates that the high enrichment of secondary metabolism genes in nonsyntenic blocks was conserved among various species for at least the 10 fungal species used in this study. However, SMB gene clusters producing common products in phylogenetically close species may often be syntenic, as previously shown in various reports, which has resulted in the failure of detection by comparisons of the corresponding clusters in the respective genomes. Typical examples of unsuccessful detections involved the combinations of SMB gene clusters for *A. flavus* aflatoxin and *A. nidulans* sterigmatocystin^19,20^ and *F. verticillioides* and *F. oxysporum* bikaverin cluster homologs.^21^ Similarly, horizontal transfer of a gene cluster may also result in unsuccessful detection of the cluster, even between species with large phylogenetic distances.^19,21^ An SMB gene cluster is known to consist of genes encoding proteins of particular characteristic functions, such as PKSs, NRPSs, Zn(II)_2_-Cys_6_ transcription factors,^22^ and major facilitator superfamily (MFS) transporters.^23^ Significant enrichment of these genes allowed identification of SMB gene clusters, owing to the overall similarity among various clusters producing different compounds and between species with large phylogenetic distances. Our method, which first detected seeds by local gene alignments and successively corrected their boundaries using simple similarity searches independent of synteny, identified SMB gene clusters more efficiently than expected prior to this study, even though nonsyntenic blocks are known to have high diversity.^24,25^

The previously reported methods predicted SMB gene clusters based on the sequence similarity of the core genes in the cluster, such as NRPS, PKS, a hybrid NRPS-PKS enzyme and DMAT.^6,7,18^ In contrast to these methods, our method does not depend on the presence of core genes. Due to this remarkable feature, the *A. oryzae* kojic acid biosynthesis gene cluster, which does not include core genes, was successfully predicted using this method. In contrast, there are also examples of missing predictions of known short SMB gene clusters, such as those responsible for the biosynthesis of asperthecin in *A. nidulans* and fusaric acid in *F. verticillioides* (Table 1). The inability to identify the gene clusters named above was due to the existence of an inversion in the former cluster and unique genes in the latter one, resulting in the failure of the local alignment of homologous gene pairs. In both cases, the short sizes of the clusters (three to five genes) prevented the remaining portions of the clusters from being identified. Similarly, intervention of a cluster by a horizontal gene transfer of another cluster (more than five genes), dividing the cluster into small segments,^26^ may also cause detection failure.

In this study, many known SMB gene clusters were identified within 19 genes as errors for the maximum CB score, when error is defined as the sum of absolute differences of cluster margins at both ends. Because our method does not depend on gene order within the length of the cluster size for the correction of cluster boundaries, this observation strongly suggests that the genes inside and outside of the clusters have different sequence characteristics. Accordingly, the probability of homology between the genes inside the clusters from the two genomes is significantly higher than (i) the probability of homology between the genes outside the clusters or (ii) the probability of homology between the genes inside the clusters and the genes outside the clusters (*P* = 6.2 × 10–121, χ^2^ test). In contrast, the cluster sizes of some gene clusters, e.g. the kojic acid biosynthesis gene cluster, were overestimated. This overestimation suggests two possibilities: (i) the two clusters were located side by side with few or no non-SMB genes in between or (ii) the gene cluster may be a part of the ancestral SMB gene cluster, with the remainder of the genes being presently inactive. In the cases of clusters with errors larger than 10 at the maximum CB value, such as the clusters for aflatoxin, terrequinone and pseurotin biosynthesis as well as kojic acid biosynthesis in Table 1, genes with characteristics of SMB genes were identified beyond the experimentally determined cluster margins.

As described above, our method is a useful means to predict SMB gene clusters, particularly novel clusters without core genes; thus, this method has the potential to discover novel mechanisms of unknown SMBs. Two major problems of our method are that short gene clusters might not be detected in some cases and that the prediction of a cluster boundary might not always be accurate. Recently, a method for predicting accurate margins of SMB gene clusters by analyzing the co-expression of neighboring genes has been reported,^27^ with the condition that the gene indispensable for the SMB gene cluster is identified using the known sequence of the core gene. Therefore, the combination of our method and the expression analysis method described above could effectively compensate for the problems that currently exist in both methods. However, our method is essentially not applicable to SMB gene clusters that are unique to particular genomes. Nevertheless, of the 24 known SMB gene clusters on the 10 genomes used in this study, 21 clusters were identified via comprehensive pairwise comparisons. The problem of detecting ‘rare SMB gene clusters’ could be solved by increasing the number of genomes used for predictions. The acceleration of sequence accumulation due to the rapid development of sequencing technologies is expected to significantly increase our method's performance of in a short period of time. A comprehensive analysis of the distribution of secondary metabolism genes and motifs in translated polypeptides across diverse species, together with the structural analyses of corresponding compounds, will open a new era in the study of secondary metabolism.

### 3.4. Availability

We intend to provide the present method as a web service (http://www.fung-metb.net/).

## Supplementary data

Supplementary Data are available at www.dnaresearch.oxfordjournals.org.

## Funding

This work was partly supported by the commission for the Development of Artificial Gene Synthesis Technology for Creating Innovative Biomaterial from the Ministry of Economy, Trade and Industry (METI), Japan.

## Supplementary Material

Supplementary Data
