# Supplementary material for: Motif-Independent Prediction of a Secondary Metabolism Gene Cluster Using Comparative Genomics: Application to Sequenced Genomes of Aspergillus and Ten Other Filamentous Fungal Species
Source: DNA Res. 2014 Apr 11;21(4):447–57. doi: 10.1093/dnares/dsu010 (PMC4131838; doi:10.1093/dnares/dsu010)
Supplement: Supplementary Data [file supp_21_4_447__index.html]

Motif-Independent Prediction of a Secondary Metabolism Gene Cluster Using Comparative Genomics: Application to Sequenced Genomes of Aspergillus and Ten Other Filamentous Fungal Species — Motif-Independent Prediction of a Secondary Metabolism Gene Cluster Using Comparative Genomics: Application to Sequenced Genomes of Aspergillus and Ten Other Filamentous Fungal Species — Supplementary Data 

# Motif-Independent Prediction of a Secondary Metabolism Gene Cluster Using Comparative Genomics: Application to Sequenced Genomes of *Aspergillus* and Ten Other Filamentous Fungal Species

## Supplementary Data

Supplementary Data

**Files in this Data Supplement:**

- Supplementary Data - Doc file
- Supplementary Figures - pptx file
- Supplementary Table 1 - doc file
- Supplementary Table 2 - xls file
- Supplementary Table 3 - xls file
- Supplementary Table 4 - xls file
- Supplementary Table 5 - xls file
