# Supplementary material for: Motif-Independent Prediction of a Secondary Metabolism Gene Cluster Using Comparative Genomics: Application to Sequenced Genomes of Aspergillus and Ten Other Filamentous Fungal Species
Source: DNA Res. 2014 Apr 11;21(4):447–57. doi: 10.1093/dnares/dsu010 (PMC4131838; doi:10.1093/dnares/dsu010)
Supplement: Supplementary Data [file supp_dsu010_dsu010supp.doc]

**Supplementary Figure S1. Detailed workflow of the method for predicting SMB gene clusters.** Cell score 0 & retraceback: To detect other seeds (R_k_, k=1, 2, 3, …) with maximum scores lower than that of R_0_ using the same score matrix, cells corresponding to R_0_ in the matrix were filled with 0 (Supplementary Fig. 1).


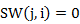
, where
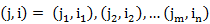


Another traceback was then performed from the cell with the maximum score that was greater than 1 to the cell with a score of 0 in the same manner as above. The gene cluster coordinate was designated R_1_ (Supplementary Fig. 1).


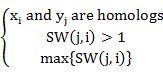
 … *1

R_k_s (k=2, 3, 4, …) was identified by repeating the above procedure until no more cells satisfying the expression *1 could be obtained. Alignment_2~: R_k_s (k=1, 2, 3, …), located close to the previously identified seeds in the matrix, might be inaccurately identified by the scores remaining in those cells close to the previously identified seeds. To reduce the influence, R_k_, containing more than 3 homologous gene pairs, was re-aligned using a (j_m_–j_1_+2)×(i_n_–i_1_+2) score matrix. Then, the resulting R_k_ was re-defined as R_kl_ (l=0, 1, 2, 3, …) (Supplementary Fig. 1). Similarly, R_0_, R_k0_, R_kl0_… were identified by repeating the same process as above until no more cells satisfying the expression *1 could be detected. Consequently, R_0_, R_k0_, R_kl0_… were obtained as a series of gene cluster coordinates identified by the first traceback in each score matrix.
