# Supplementary material for: Motif-Independent Prediction of a Secondary Metabolism Gene Cluster Using Comparative Genomics: Application to Sequenced Genomes of Aspergillus and Ten Other Filamentous Fungal Species
Source: DNA Res. 2014 Apr 11;21(4):447–57. doi: 10.1093/dnares/dsu010 (PMC4131838; doi:10.1093/dnares/dsu010)
Supplement: Supplementary Data [file supp_dsu010_dsu010supp_figs.pptx]

## Slide 1
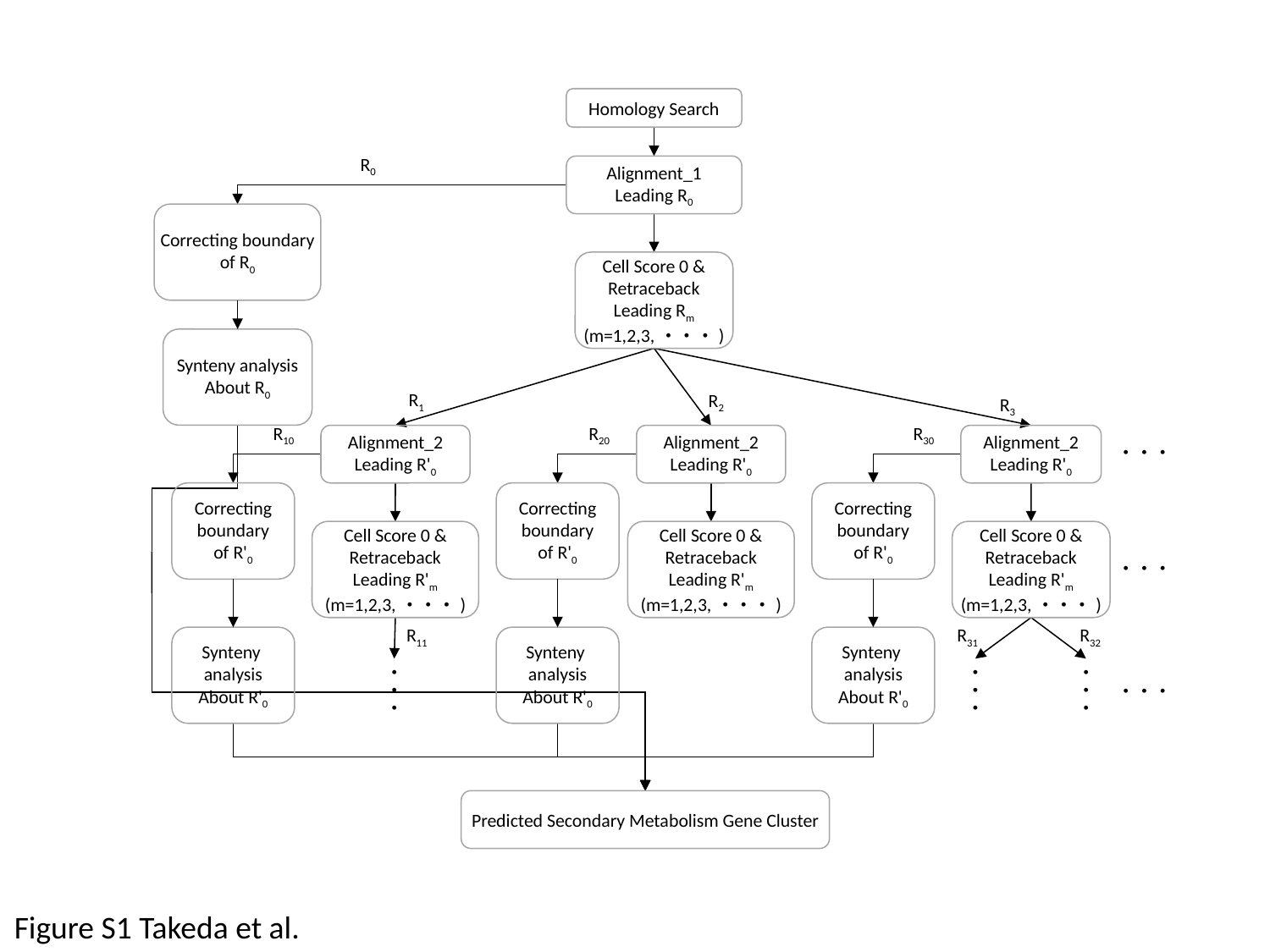

Homology Search
R0
Alignment_1
Leading R0
Correcting boundary
of R0
Cell Score 0 &
Retraceback
Leading Rm
(m=1,2,3,・・・)
Synteny analysis
About R0
R1
R2
R3
R10
R20
R30
Alignment_2
Leading R'0
Alignment_2
Leading R'0
Alignment_2
Leading R'0
・・・
Correcting
boundary
of R'0
Correcting
boundary
of R'0
Correcting
boundary
of R'0
Cell Score 0 &
Retraceback
Leading R'm
(m=1,2,3,・・・)
Cell Score 0 &
Retraceback
Leading R'm
(m=1,2,3,・・・)
Cell Score 0 &
Retraceback
Leading R'm
(m=1,2,3,・・・)
・・・
R11
R31
R32
Synteny
analysis
About R'0
Synteny
analysis
About R'0
Synteny
analysis
About R'0
・
・
・
・
・
・
・
・
・
・・・
Predicted Secondary Metabolism Gene Cluster
Figure S1 Takeda et al.
