# Supplementary material for: Motif-Independent Prediction of a Secondary Metabolism Gene Cluster Using Comparative Genomics: Application to Sequenced Genomes of Aspergillus and Ten Other Filamentous Fungal Species
Source: DNA Res. 2014 Apr 11;21(4):447–57. doi: 10.1093/dnares/dsu010 (PMC4131838; doi:10.1093/dnares/dsu010)
Supplement: Supplementary Data [file supp_dsu010_dsu010supp_table1.doc]

**Supplementary Table S1.** Experimentally characterized clusters

| Product | Experimentally characterized clusters | | Reference |
| --- | --- | --- | --- |
|  | Begin | End |  |
| Aflatoxin | AFLA_139150 | AFLA_139440 | Yu et al.[^1^](#_ENREF_21),[^2^](#_ENREF_22) |
| Gliotoxin | AFLA_064330 | AFLA_064650 | Hypothetical |
| Kojic acid | AO090113000136 | AO090113000138 | Terabayashi et al.[^3^](#_ENREF_10) |
| Asperfuranone | ANID_01036 | ANID_01029 | Chiang et al.[^4^](#_ENREF_23) |
| Asperthecin | ANID_06002 | ANID_06000 | Szewczyk et al.[^5^](#_ENREF_24) |
| Penicillin | ANID_02625 | ANID_02621 | Brakhage et al.[^6^](#_ENREF_25) |
| Sterigmatocystin | ANID_07804 | ANID_07825 | Brown et al.[^7^](#_ENREF_26) |
| Terrequinone | ANID_08513 | ANID_08519 | Bouhired el al.[^8^](#_ENREF_27) |
| Ergot | Afu2g17960 | Afu2g18060 | Coyle et al.[^9^](#_ENREF_28), Unsold et al. |
| ETP^a^ | Afu3g12890 | Afu3g12960 | Hypothetical |
| Fumitremorgin | Afu8g00170 | Afu8g00260 | Maiya et al.[^12^](#_ENREF_31); Grundmann et al.[^13^](#_ENREF_31); Kato et al.[^14^](#_ENREF_32) |
| Gliotoxin | Afu6g09630 | Afu6g09740 | Gardiner & Howlett[^15^](#_ENREF_33) |
| Melanin | Afu2g17530 | Afu2g17600 | Fujii et al.[^16^](#_ENREF_34); Tsai et al.[^17^](#_ENREF_35) |
| Pes1 | Afu1g10380 | Afu1g10390 | Perrin et al.[^18^](#_ENREF_36) |
| Pseurotin | Afu8g00530 | Afu8g00580 | Maiya et al.[^19^](#_ENREF_37) |
| Siderophore | Afu3g03350 | Afu3g03470 | Reiber et al.[^20^](#_ENREF_38) |
| Lovastatin | ATEG_09958 | ATEG_09974 | Kennedy et al.[^21^](#_ENREF_39); Hendrickson et al.[^22^](#_ENREF_40) |
| Furofusarin | FGSG_02320 | FGSG_02330 | Malz et al.[^23^](#_ENREF_41) |
| Fearalenone | FGSG_02395 | FGSG_02398 | Kim et al.[^24^](#_ENREF_42) |
| Fikaverin | FVEG_03384 | FVEG_03379 | Brown et al.[^25^](#_ENREF_43) |
| Fumonisin | FVEG_00329 | FVEG_00315 | Proctor et al.[^26^](#_ENREF_44) |
| Fusaric acid | FVEG_12523 | FVEG_12519 | Brown et al.[^27^](#_ENREF_45) |
| Fusarin | FVEG_11078 | FVEG_11086 | Brown et al.[^27^](#_ENREF_45) |
| Perithecium pigment | FVEG_03700 | FVEG_03695 | Brown et al.[^27^](#_ENREF_45); Proctor et al.[^28^](#_ENREF_46) |

^a^ Epipolythiodioxopiperazine type toxin.

Reference

1. Yu, J., Ronning, C. M., Wilkinson, J. R., et al. 2007, Gene profiling for studying the mechanism of aflatoxin biosynthesis in Aspergillus flavus and A. parasiticus. *Food Addit Contam*, **24**, 1035-1042.

2. Yu, J., Chang, P. K., Ehrlich, K. C., et al. 2004, Clustered pathway genes in aflatoxin biosynthesis. *Appl Environ Microbiol*, **70**, 1253-1262.

3. Terabayashi, Y., Sano, M., Yamane, N., et al. 2010, Identification and characterization of genes responsible for biosynthesis of kojic acid, an industrially important compound from Aspergillus oryzae. *Fungal Genet Biol*, **47**, 953-961.

4. Chiang, Y. M., Szewczyk, E., Davidson, A. D., Keller, N., Oakley, B. R. and Wang, C. C. 2009, A gene cluster containing two fungal polyketide synthases encodes the biosynthetic pathway for a polyketide, asperfuranone, in Aspergillus nidulans. *J Am Chem Soc*, **131**, 2965-2970.

5. Bok, J. W., Chiang, Y. M., Szewczyk, E., et al. 2009, Chromatin-level regulation of biosynthetic gene clusters. *Nat.Chem.Biol.*, **5**, 462-464.

6. Brakhage, A. A., Al-Abdallah, Q., Tuncher, A. and Sprote, P. 2005, Evolution of beta-lactam biosynthesis genes and recruitment of trans-acting factors. *Phytochemistry*, **66**, 1200-1210.

7. Brown, D. W., Yu, J. H., Kelkar, H. S., et al. 1996, Twenty-five coregulated transcripts define a sterigmatocystin gene cluster in Aspergillus nidulans. *Proc Natl Acad Sci U S A*, **93**, 1418-1422.

8. Bouhired, S., Weber, M., Kempf-Sontag, A., Keller, N. P. and Hoffmeister, D. 2007, Accurate prediction of the Aspergillus nidulans terrequinone gene cluster boundaries using the transcriptional regulator LaeA. *Fungal Genet Biol*, **44**, 1134-1145.

9. Coyle, C. M. and Panaccione, D. G. 2005, An ergot alkaloid biosynthesis gene and clustered hypothetical genes from Aspergillus fumigatus. *Appl Environ Microbiol*, **71**, 3112-3118.

10. Unsold, I. A. and Li, S. M. 2006, Reverse prenyltransferase in the biosynthesis of fumigaclavine C in Aspergillus fumigatus: gene expression, purification, and characterization of fumigaclavine C synthase FGAPT1. *Chembiochem*, **7**, 158-164.

11. Unsold, I. A. and Li, S. M. 2005, Overproduction, purification and characterization of FgaPT2, a dimethylallyltryptophan synthase from Aspergillus fumigatus. *Microbiology*, **151**, 1499-1505.

12. Maiya, S., Grundmann, A., Li, S. M. and Turner, G. 2006, The fumitremorgin gene cluster of Aspergillus fumigatus: identification of a gene encoding brevianamide F synthetase. *Chembiochem*, **7**, 1062-1069.

13. Grundmann, A., Kuznetsova, T., Afiyatullov, S. and Li, S. M. 2008, FtmPT2, an N-prenyltransferase from Aspergillus fumigatus, catalyses the last step in the biosynthesis of fumitremorgin B. *Chembiochem*, **9**, 2059-2063.

14. Kato, N., Suzuki, H., Takagi, H., et al. 2009, Identification of cytochrome P450s required for fumitremorgin biosynthesis in Aspergillus fumigatus. *Chembiochem*, **10**, 920-928.

15. Gardiner, D. M. and Howlett, B. J. 2005, Bioinformatic and expression analysis of the putative gliotoxin biosynthetic gene cluster of Aspergillus fumigatus. *FEMS Microbiol Lett*, **248**, 241-248.

16. Fujii, T., Yamaoka, H., Gomi, K., Kitamoto, K. and Kumagai, C. 1995, Cloning and nucleotide sequence of the ribonuclease T1 gene ( rntA ) from Aspergillus oryzae and its expression in Saccharomyces cerevisiae and Aspergillus oryzae *Biosci.Biotechnol.Biochem.*, **59**, 1869-1874.

17. Tsai, H. F., Wheeler, M. H., Chang, Y. C. and Kwon-Chung, K. J. 1999, A developmentally regulated gene cluster involved in conidial pigment biosynthesis in Aspergillus fumigatus. *J Bacteriol*, **181**, 6469-6477.

18. Perrin, R. M., Fedorova, N. D., Bok, J. W., et al. 2007, Transcriptional regulation of chemical diversity in Aspergillus fumigatus by LaeA. *PLoS Pathog*, **3**, e50.

19. Maiya, S., Grundmann, A., Li, X., Li, S. M. and Turner, G. 2007, Identification of a hybrid PKS/NRPS required for pseurotin A biosynthesis in the human pathogen Aspergillus fumigatus. *Chembiochem*, **8**, 1736-1743.

20. Reiber, K., Reeves, E. P., Neville, C. M., et al. 2005, The expression of selected non-ribosomal peptide synthetases in Aspergillus fumigatus is controlled by the availability of free iron. *FEMS Microbiol Lett*, **248**, 83-91.

21. Kennedy, J., Auclair, K., Kendrew, S. G., Park, C., Vederas, J. C. and Hutchinson, C. R. 1999, Modulation of polyketide synthase activity by accessory proteins during lovastatin biosynthesis. *Science*, **284**, 1368-1372.

22. Hendrickson, L., Davis, C. R., Roach, C., et al. 1999, Lovastatin biosynthesis in Aspergillus terreus: characterization of blocked mutants, enzyme activities and a multifunctional polyketide synthase gene. *Chem Biol*, **6**, 429-439.

23. Malz, S., Grell, M. N., Thrane, C., et al. 2005, Identification of a gene cluster responsible for the biosynthesis of aurofusarin in the Fusarium graminearum species complex. *Fungal Genet Biol*, **42**, 420-433.

24. Kim, Y. T., Lee, Y. R., Jin, J., et al. 2005, Two different polyketide synthase genes are required for synthesis of zearalenone in Gibberella zeae. *Mol Microbiol*, **58**, 1102-1113.

25. Brown, D. W., Butchko, R. A. and Proctor, R. H. 2008, Genomic analysis of Fusarium verticillioides. *Food Addit Contam Part A Chem Anal Control Expo Risk Assess*, **25**, 1158-1165.

26. Proctor, R. H., Brown, D. W., Plattner, R. D. and Desjardins, A. E. 2003, Co-expression of 15 contiguous genes delineates a fumonisin biosynthetic gene cluster in Gibberella moniliformis. *Fungal Genet Biol*, **38**, 237-249.

27. Brown, D. W., Butchko, R. A., Busman, M. and Proctor, R. H. 2012, Identification of gene clusters associated with fusaric acid, fusarin, and perithecial pigment production in Fusarium verticillioides. *Fungal Genet Biol*, **49**, 521-532.

28. Proctor, R. H., Butchko, R. A., Brown, D. W. and Moretti, A. 2007, Functional characterization, sequence comparisons and distribution of a polyketide synthase gene required for perithecial pigmentation in some Fusarium species. *Food Addit Contam*, **24**, 1076-1087.
